# Paracrine stimulation of perinatal lung functional and structural maturation by mesenchymal stem cells

**DOI:** 10.1186/s13287-020-02028-4

**Published:** 2020-12-09

**Authors:** Janine Obendorf, Claire Fabian, Ulrich H. Thome, Mandy Laube

**Affiliations:** 1 Center for Pediatric Research Leipzig, Department of Pediatrics, Division of Neonatology, University of Leipzig, Liebigstrasse 19, 04103 Leipzig, Germany; 2 Fraunhofer Institute for Cell Therapy and Immunology, Perlickstrasse 1, 04103 Leipzig, Germany

**Keywords:** Mesenchymal stem cells, Fetal lung maturation, Epithelial sodium channel, Na^+^ transport, Preterm infants, Respiratory distress

## Abstract

**Background:**

Mesenchymal stem cells (MSCs) were shown to harbor therapeutic potential in models of respiratory diseases, such as bronchopulmonary dysplasia (BPD), the most common sequel of preterm birth. In these studies, cells or animals were challenged with hyperoxia or other injury-inducing agents. However, little is known about the effect of MSCs on immature fetal lungs and whether MSCs are able to improve lung maturity, which may alleviate lung developmental arrest in BPD.

**Methods:**

We aimed to determine if the conditioned medium (CM) of MSCs stimulates functional and structural lung maturation. As a measure of functional maturation, Na^+^ transport in primary fetal distal lung epithelial cells (FDLE) was studied in Ussing chambers. Na^+^ transporter and surfactant protein mRNA expression was determined by qRT-PCR. Structural maturation was assessed by microscopy in fetal rat lung explants.

**Results:**

MSC-CM strongly increased the activity of the epithelial Na^+^ channel (ENaC) and the Na,K-ATPase as well as their mRNA expression. Branching and growth of fetal lung explants and surfactant protein mRNA expression were enhanced by MSC-CM. Epithelial integrity and metabolic activity of FDLE cells were not influenced by MSC-CM. Since MSC’s actions are mainly attributed to paracrine signaling, prominent lung growth factors were blocked. None of the tested growth factors (VEGF, BMP, PDGF, EGF, TGF-β, FGF, HGF) contributed to the MSC-induced increase of Na^+^ transport. In contrast, inhibition of PI3-K/AKT and Rac1 signaling reduced MSC-CM efficacy, suggesting an involvement of these pathways in the MSC-CM-induced Na^+^ transport.

**Conclusion:**

The results demonstrate that MSC-CM strongly stimulated functional and structural maturation of the fetal lungs. These effects were at least partially mediated by the PI3-K/AKT and Rac1 signaling pathway. Thus, MSCs not only repair a deleterious tissue environment, but also target lung cellular immaturity itself.

## Background

Lung maturation is a complex process that is influenced by a variety of factors. Growth factor signaling as well as ion channel functions are crucially involved in intrauterine lung proliferation and differentiation. During fetal development, the lung undergoes extensive changes in fluid secretion and absorption [1]. Fluid transport across alveolar epithelial cells is mediated by Na^+^ influx through epithelial Na^+^ channels (ENaC) in the apical membrane [2]. At the basolateral side, Na^+^ is actively extruded by the Na,K-ATPase, generating an osmotic gradient that drives fluid diffusion across the alveolar epithelium [3]. Vectorial Na^+^ transport is regulated by several pathways, including steroids [4], growth factors [5, 6], and kinase signaling [7, 8]. Furthermore, the surfactant is secreted by mature alveolar type II (ATII) cells to prevent alveolar collapse during end-expiration [9]. Disruption of Na^+^ transport-driven alveolar fluid clearance (AFC) and insufficient surfactant secretion due to preterm birth can lead to serious pulmonary complications and respiratory distress.

Among them, bronchopulmonary dysplasia (BPD) is a severe neonatal chronic lung disease first described by Northway and colleagues in 1967 [10]. The so-called "old" BPD was characterized by intense inflammation, fibrosis, and lung injury due to disruption of pulmonary structures [11]. In contrast, advances in neonatal care (surfactant therapy, optimized ventilation) and survival of extremely preterm infants have changed the disease pathology [12]. The “new” BPD is characterized predominantly by simplified lung structure with fewer and larger alveoli, especially in extremely preterm infants born at earlier gestational ages (24–27 weeks) [12, 13]. BPD is a multifactorial disease and a variety of therapeutic strategies have been exerted; however, current treatment options are mainly palliative, and until now, no promising pharmacological approach exists to prevent or cure the disease [14].

An increased interest in stem cells has emerged in recent years. Mesenchymal stem cells (MSCs) are multipotent cells found in a variety of adult tissues, such as the adipose tissue, bone marrow, amniotic fluid, placenta, or umbilical cord, the latter containing MSCs in the cord lining membrane as well as in Wharton’s jelly [15, 16]. The efficacy of cell-based therapies is mainly attributed to paracrine signaling [17]. MSCs secrete immunomodulatory factors and several growth factors including fibroblast growth factor (FGF), hepatocyte growth factor (HGF), epidermal growth factor (EGF), and vascular endothelial growth factor (VEGF) [18]. MSC-based studies have shown beneficial effects in animal models of BPD. Administration of bone marrow (BM)-derived MSCs and its conditioned medium for example ameliorated parenchymal and vascular injury in neonatal mice exposed to hyperoxia [19]. Furthermore, BM-derived MSCs lowered pulmonary hypertension, improved survival, and preserved alveolar and vascular growth in hyperoxia-exposed rat pups [20]. MSCs also stimulated branching morphogenesis of fetal rat lung explants [21] and increased lung organoid formation and alveolar differentiation in 3D progenitor cell cultures [22]. Thus, the in vitro and in vivo studies showed promising results of MSC application, but focused mainly on the repair of disease pathways by counteracting inflammation and oxygen toxicity. In contrast, few studies have investigated the effect of MSCs on lung maturation. It is yet unknown whether or not MSCs stimulate lung maturation in immature fetal lungs prior to disease onset. Since improving lung immaturity may be an effective strategy to enhance lung development and function in preterm infants, the aim of the present study was to elucidate maturational responses of fetal distal lung epithelial cells (FDLE) exposed to conditioned medium of MSCs (MSC-CM). Epithelial Na^+^ transport and structural maturation of fetal lung explants were studied as markers of maturation. Furthermore, the involved pathways of MSC’s effects were investigated by inhibiting several growth factor receptors and kinases. Our findings support the hypothesis that MSCs constitute a therapeutic option for respiratory distress in preterm infants by elevating AFC and structural lung maturation involving kinase and Rho-GTPase signaling.

## Methods

### Cell isolation and culture

#### Isolation and characterization of human MSCs

The study was approved by the ethical board of the medical faculty of Leipzig University. The umbilical cord (UC) tissue was collected after delivery from newborns whose mothers granted informed consent. MSC isolation and characterization are described in the Supplements.

#### Isolation of FDLE cells

All animal care and experimental procedures were approved by the institutional review board (Landesdirektion Leipzig, Permit number: T23/15). Sprague-Dawley rats were bred at the Medical Experimental Center (MEZ) of Leipzig University and housed in rooms with a controlled temperature of 22 °C, 55% humidity, and a 12-h light-dark cycle. Food and water were freely available. Rats were euthanized by Pentobarbital injection.

FDLE cells were isolated at gestational day 19 to 20 from rat fetuses as described elsewhere [23–25]. Briefly, the fetal lungs were minced and digested in Hanks’ Balanced Salt Solution (Life Technologies, Carlsbad, USA), containing 0.125% trypsin (Life Technologies), and 0.4 mg/ml DNAse (Merck, Darmstadt, Germany) for 10 min at 37 °C. Digestion was stopped with MEM (Life Technologies) supplemented with 10% FBS. The cell suspension was centrifuged twice and the pellet resuspended in MEM. After centrifugation, cells were resuspended in MEM containing 0.1% collagenase type I (CellSystems, Troisdorf, Germany) and 0.2 mg/ml DNAse and incubated for 15 min at 37 °C. After adding MEM with 10% FBS (Biochrom, Berlin, Germany), the cell suspension was centrifuged and resuspended in MEM containing 10% FBS, and plated twice in cell culture flasks at 37 °C for 1 h to remove contaminating fibroblasts. For Ussing chamber studies, the cells were seeded on permeable Snapwell inserts with a surface area of 1.1 cm^2^ (Costar, #3407, Corning Inc., NY, USA) at a density of 10^6^ per insert. For RNA isolation, the cells were seeded on cell culture inserts with a surface area of 4.6 cm^2^ (ThinCert, #657641, Greiner Bio-One, Frickenhausen, Germany) at a density of 2 × 10^6^ cells per insert. FDLE cells were cultured submerged in MEM with 10% FBS, l-glutamine (2 mM, Merck), penicillin, streptomycin, and amphotericin B, with daily media exchange. 24 h prior to measurements the FDLE cell medium was either replaced by MSC-CM or control medium, containing DMEM (low glucose, GlutaMAX™) with 2% FBS, accordingly. For receptor tyrosine kinase (RTK) inhibition, the respective inhibitor was added to the culture medium 24 h before analysis. The inhibitors of the VEGF receptor (Axitinib, 10 μM), the bone morphogenetic protein receptor (BMP-R; K02288, 1 μM), the platelet-derived growth factor receptor (PDGF-R; AG-1296, 10 μM), the EGF receptor (EGF-R; AG-1478, 1 μM), the transforming growth factor β receptor (TGF-β-R; SB431542, 1 μM), the FGF receptor (FGF-R; FIIN-2, 1 μM), and the HGF receptor c-met (PHA665752, 1 μM) were purchased from Biomol (Hamburg, Germany). The inhibitor of the Ras-related C3 botulinum toxin substrate 1 (Rac1; Ehop-016, 1 μM) was obtained from Selleckchem (Houston, USA). The inhibitors of the phosphoinositide 3-kinase (PI3-K; LY294002, 10 μM) and the protein kinase B (AKT; Akti 1/2, 10 μM) were purchased from Merck. Inhibitors were dissolved in DMSO, and the respective controls were treated with the solvent to exclude any solvent influence on the evoked responses.

#### Fetal lung isolation for explant culture

Fetal rat lungs were isolated at embryonic day (ED) 15 of gestation from Sprague-Dawley fetuses according to the protocol of del Moral et al. [26]. For explant cultivation, the lungs were incubated on cell culture inserts at an air-liquid-interface for an additional 4 days. The culture medium was changed daily, and growth was documented with a Zeiss Axio Zoom.V16 Stereo Microscope (Zeiss, Oberkochen, Germany). Every experiment was repeated at least three times with a minimum of three lungs per group. To calculate total lung explant and tissue area, microscopic pictures of the whole lungs, or hematoxylin and eosin (HE)-stained whole lung slices were analyzed with ImageJ software (National Institutes of Health, Bethesda, USA). ImageJ macros used for area analysis are illustrated in the Supplements. The number of peripheral airway buds was counted on the day of isolation (ED15) and 2 days later (ED15 + 2). In addition, the ratio of the increase in airway bud number from ED15 to ED15 + 2 was calculated, as described elsewhere [27].

### Immunofluorescence staining of fetal lung explants

For immunofluorescence staining, lung explants were fixed with 4% formaldehyde in PBS for 2 h at 4 °C. Upon fixation, frozen sections of 6 μm were cut and slides stored at − 20 °C. After treatment with blocking solution (5% BSA in 0.3% Triton X-100/PBS) for 1 h, lung explant slices were incubated with rabbit anti-epithelial cell adhesion molecule (EpCAM) primary antibody (1:50 in blocking solution; Abcam, Cambridge, UK) at 4 °C overnight. The secondary antibody anti-rabbit (Northern Lights™ NL493 conjugated, 1:200 in blocking solution; R&D Systems, Boston, USA) was applied for 2 h. Nuclei were stained with DAPI (1 μg/ml). Immunofluorescence staining was analyzed with confocal laser-scanning microscopy (LSM710; Zeiss).

### Cell permeability assay

To assess epithelial permeability, FDLE cells were cultivated on Snapwell inserts. After 2 days of culture, the medium was changed to MSC-CM or control medium for 24 h. Afterwards, the medium was replaced by phenol red-free MEM (Life Technologies) in the lower compartment and phenol red-free MEM containing FITC-dextran (0.25 mg/ml, 3–5 kDa, Merck) in the upper compartment. After incubation of cells at 37 °C for another 24 h, the medium of the lower compartment was analyzed for fluorescence intensity of FITC-dextran. A standard dilution series of FITC-dextran was used for quantification.

### Ussing chamber measurements

All Ussing chamber experiments were performed 4 days after seeding FDLE on Snapwell inserts with an EM-CSYS-8 EasyMount Ussing chamber system (Physiological Instruments, San Diego, CA, USA) at 37 °C and continuous gas supply with 5% CO_2_ and 95% O_2_. Ussing chambers were filled with Ringer solution containing: 145 mM Na^+^, 5 mM K^+^, 1.2 mM Ca^2+^, 1.2 mM Mg^2+^, 125 mM Cl^−^, 25 mM HCO_3_^−^, 3.3 mM H_2_PO_4_^−^, and 0.8 mM HPO_4_^2−^ (pH 7.4). On the basolateral side, 10 mM glucose and on the apical side 10 mM mannitol were used. Measurements with a transepithelial resistance (*R*_te_) below 300 Ω*cm^2^ were excluded from analyses. Equivalent short-circuit currents (*I*_SC_) were assessed every 20 s by measuring transepithelial potential difference (*V*_te_) and *R*_te_ with a transepithelial current clamp and calculating the quotient *I*_SC_=*V*_te_/*R*_te_. After the baseline *I*_SC_ reached a stable plateau (*I*_base_), amiloride (10 μM, # A7410, Merck) was added to the apical compartment to determine the amiloride-sensitive ∆*I*_SC_ (∆*I*_amil_).

To determine the maximal ENaC activity (*amil*_max_), 140 mM of basolateral Na^+^ was replaced by 116 mM *N*-methyl-d-glucamine (NMDG^+^, # M-2004, Sigma-Aldrich) and 24 mM choline, generating a 145:5 apical-to-basolateral Na^+^ gradient. Then, the *I*_SC_ was measured every 5 s with a transepithelial voltage clamp. The basolateral membrane was permeabilized with amphotericin B (100 μM, Merck), uncoupling the Na,K-ATPase. When the maximum current increase was reached, amiloride was added to the apical compartment to determine *amil*_max_. On the other hand, *ouab*_max_ represents the maximum activity of the Na,K-ATPase. To determine *ouab*_max_, the apical membrane was permeabilized with amphotericin B (10 μM). When the *I*_SC_ had reached its maximum value, the Na,K-ATPase inhibitor ouabain (1 mM, Merck) was added to the basolateral compartment. The ouabain-sensitive component of the amphotericin-induced maximal *I*_SC_ (*ouab*_max_) was calculated, accordingly.

### Gene expression analyses

Total RNA was isolated 5 days after seeding of the FDLE cells using the PureLink™ RNA Kit (ThermoFisher) according to the manufacturers’ protocol. For elimination of genomic DNA and cDNA synthesis, the Maxima H Minus First-Strand cDNA Synthesis Kit, with dsDNase (ThermoFisher), was used. The total RNA (1 μg) was reversely transcribed and cDNA further diluted 1:10 in Tris-EDTA (Applichem). Real-time quantitative PCR (qPCR) analyses were conducted with the CFX Connect™ Real-Time PCR Detection System (BioRad, Hercules, USA) using SYBR™ Select Mastermix for CFX (ThermoFisher) and gene-specific primers [4, 28]. In each qPCR run, at least three biological and four technical replicates were analyzed. Serial dilutions of target-specific plasmid DNA were used for absolute quantification. Target gene expression was normalized using the reference gene mitochondrial ribosomal protein S18a (*Mrps18a*). Constant expression of *Mrps18a* was confirmed against other common reference genes.

### MTT

Metabolic activity in FDLE cells was determined with 3-(4,5-dimethylthiazol-2-yl)-2,5-diphenyltetrazolium bromide (MTT) assays. To this end, the medium was changed to a culture medium containing 5 mg/ml Thiazolyl Blue Tetrazolium Bromide solution (Applichem, Darmstadt, Germany). After an incubation period of 4 h at 37 °C, the medium was discarded and the reaction stopped with a solution containing 1:1 dimethylformamide and 20% SDS (Roth, Karlsruhe, Germany). After 1 h incubation at 37 °C, formazan crystals were dissolved, and the supernatant analyzed at 570 nm.

### Statistical analysis

Differences between two groups were analyzed with the unpaired *t* test. Otherwise, significant differences were determined by a one-way ANOVA with Tukey’s post hoc test. A probability of *p* < 0.05 was considered significant for all statistical analyses. Statistical analysis was conducted using GraphPad Prism Software (version 5.03, Graph Pad software, Inc., San Diego, CA, USA).

## Results

### UC-MSC characterization

According to the minimal criteria defined by the International Society for Cellular Therapy, we analyzed the surface marker expression and tri-lineage differentiation capacity of isolated UC-MSCs to demonstrate their stem cell properties (Fig. S1). The cells strongly expressed the common MSC markers CD44 (94 ± 3.15%), CD73 (94 ± 1.39%), and CD90 (90 ± 1.88%), whereas the expression of hematopoietic markers CD11b (26 ± 3.12%) and CD45 (13 ± 2.24%) was low. Analysis of adipogenic differentiation using Oil red O staining showed a high number of small intracellular lipid droplets. Staining of acidic polysaccharides with Alcian blue showed that UC-MSCs differentiated reliably into chondrogenic cells. Osteogenic differentiation was demonstrated with ALP 1 staining, although the differentiation process was slower (4 weeks) compared to chondrogenic and adipogenic differentiation (2 weeks).

### MSC-CM induces functional maturation of FDLE cells

**Fig. 1:**
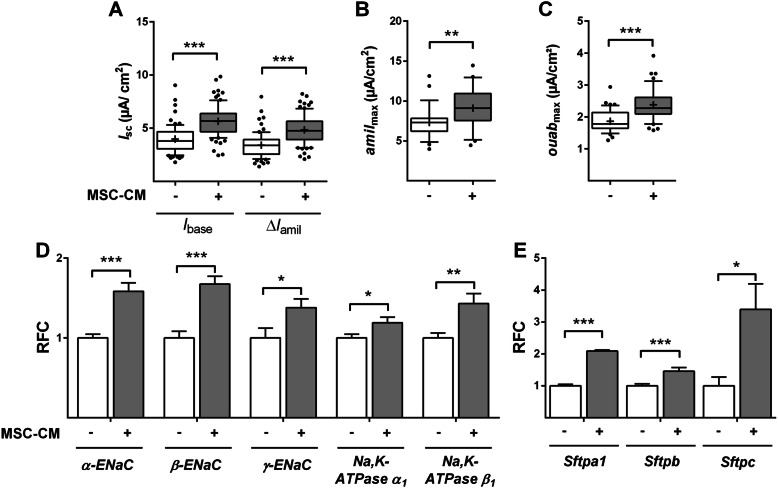
MSC-CM increased activity and mRNA expression of Na^+^ transporters in FDLE cells. FDLE cells were subjected to MSC-CM or control medium for 24 h. Statistical differences between the two groups were analyzed with a *t* test. **a**–**c** Data are displayed as boxes and whiskers with the 10–90 percentiles, mean (+) and median (horizontal line). **a** MSC-CM significantly enhanced *I*_base_ and ∆*I*_amil_ in FDLE cells (*n* = 78–82, 4 independent experiments (IE); ***p < 0.001). **b** Maximal ENaC activity (*amil*_max_) was significantly higher in FDLE cells cultured in MSC-CM (*n* = 26–27, 3 IE; **p < 0.01). **c** Maximal Na,K-ATPase activity (*ouab*_max_) was higher in FDLE cells treated with MSC-CM (*n* = 42–46, 2 IE; ***p < 0.001). **d**, **e** Data are displayed as mean + SEM. **d** mRNA expression of ENaC subunits (*ENaCα*, *ENaCβ*, *ENaCγ*) and Na,K-ATPase subunits (*Na,K-ATPase α*_*1*_ & *Na,K-ATPase β*_*1*_) were increased by MSC-CM (*n* = 6–8, 2 IE; *p < 0.05; **p < 0.01; ***p < 0.001). **e** Surfactant protein A1, B, C (*Sftpa1, Sftpb, Sftpc*) mRNA levels were elevated by MSC-CM (*n* = 3; *p < 0.05; ***p < 0.001). □ control, ■ MSC-CM

MSC-CM incubation of FDLE cells for 24 h significantly elevated *I*_base_ and Δ*I*_amil_ 1.4-fold compared with control cells (*p* < 0.001, Fig. 1a). MSC-CM further strongly increased *amil*_max_ in FDLE cells compared with control cells (*p* < 0.01, Fig. 1b). In addition, maximal Na,K-ATPase activity was strongly elevated by MSC-CM, as shown by the significantly elevated *ouab*_max_ in FDLE monolayers (*p* < 0.001, Fig. 1c). In conclusion, MSC-CM profoundly increased epithelial Na^+^ transport, as well as the maximal ENaC and maximal Na,K-ATPase activity in FDLE cells.

MSC-CM-incubated FDLE cells showed a significantly higher mRNA expression of the *α-ENaC*, *β*-*ENaC*, and *γ-ENaC* subunits compared to control cells (*p* < 0.01, *p* < 0.001, Fig. 1d). Expression of *α-ENaC* was 1.6-fold higher in MSC-CM-treated cells compared to control cells. Furthermore, mRNA expression of *β*-*ENaC* and *γ-ENaC* were 1.7-fold and 1.4-fold higher in FDLE cells cultivated in MSC-CM. In addition, mRNA expression of the *Na,K-ATPase* subunits *α*_*1*_ and *β*_*1*_ was significantly higher in FDLE cells grown in MSC-CM (*p* < 0.05, *p* < 0.01, Fig. 1d). The *Na,K-ATPase α*_*1*_ expression level was 1.2-fold higher, while MSC-CM elevated the *Na,K-ATPase β*_*1*_ expression 1.4-fold. These results demonstrate that MSC-CM increased mRNA expression of the involved Na^+^ transporters, possibly contributing to the increased Na^+^ transport shown before.

Another important feature of perinatal lung transition and a commonly used marker of lung maturation is the secretion of surfactant by ATII cells. Surfactant proteins A, B, and C mRNA expressions were significantly induced by MSC-CM (*p* < 0.05, *p* < 0.001, Fig. 1e). Expression of *Sftpa1* was 2.1-fold higher than in control cells. Furthermore, *Sftpb* was 1.5-fold higher and *Sftpc* was 3.4-fold higher in MSC-CM-treated compared to control FDLE cells. Herein, another crucial alveolar function was augmented by MSC-CM, further supporting the induction of functional maturation.

### MSC-CM enhances the structural maturation of fetal lung explants

**Fig. 2:**
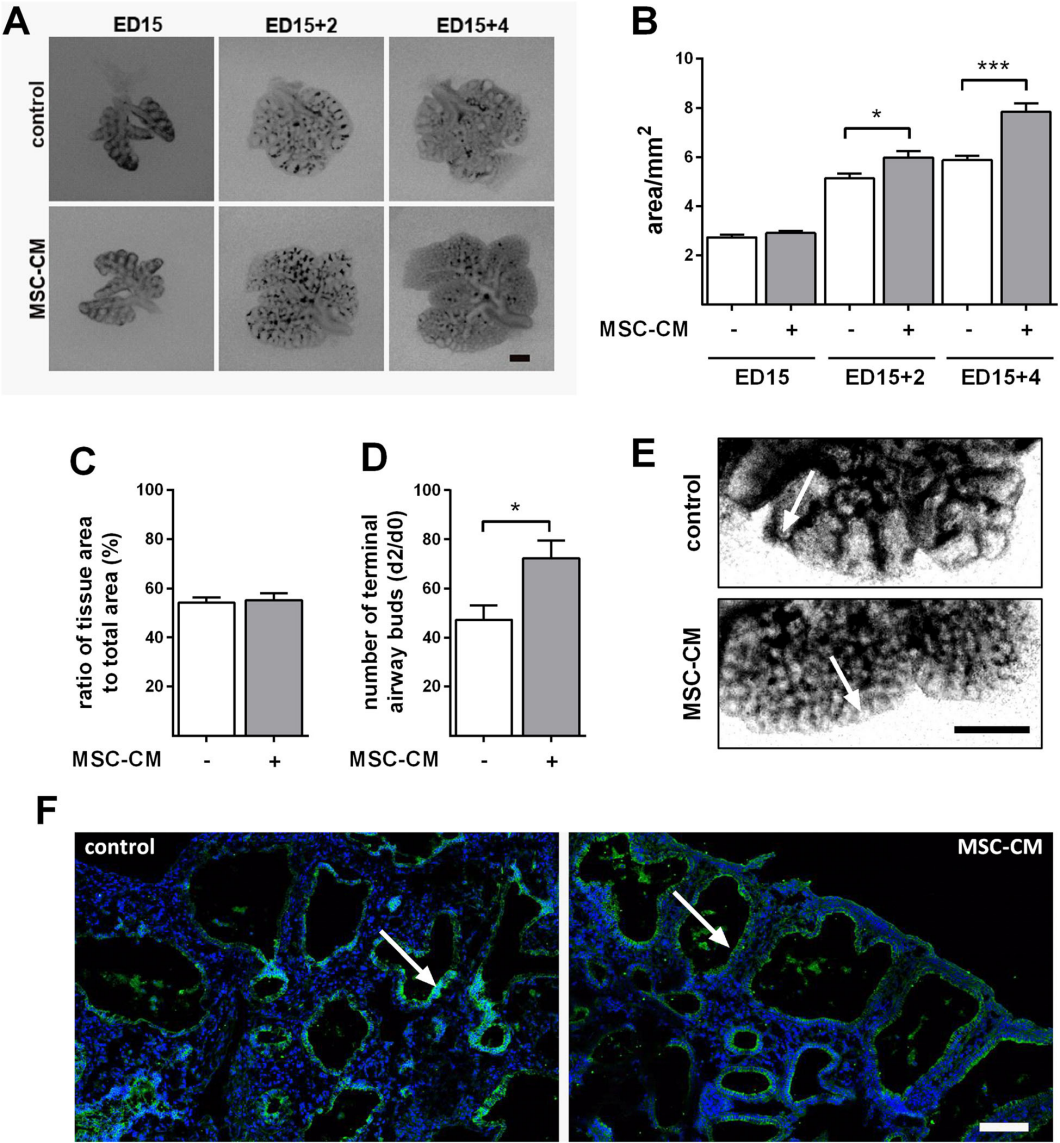
MSC-CM enhanced structural maturation and growth of fetal lung explants. Embryonic day 15 (ED15) fetal lung explants were subjected to MSC-CM or control medium for 4 days. Data are displayed as mean + SEM. Statistical differences between the two groups were analyzed with a *t* test. **a**, **b** Fetal lung explants cultured in MSC-CM were larger compared to controls (*n* = 9; *p < 0.05; ***p < 0.001; scale bar: 500 μm). **c** The ratio of tissue area to total area was similar in control and MSC-CM-treated lungs (*n* = 9). **d**, **e** MSC-CM increased the number of peripheral airway buds in lung explants (*n* = 3; *p < 0.05; scale bar: 500 μm). **f** No differences in the degree of parenchymal development were noted, as shown by EpCAM (green, arrow) and DAPI (blue, nuclei) stainings. (scale bar: 100 μm). □ control, ■ MSC-CM

Lung explants treated with MSC-CM for 4 days displayed an enhanced branching and structural morphology compared to control explants (Fig. 2a, e). The area of lung explants increased during culture in control and MSC-CM, but MSC-CM significantly raised explant area after 2 and 4 days in culture compared to control explants (*p* < 0.05, *p* < 0.001, Fig. 2a, b). To exclude that the growth surge induced by MSC-CM is the result of abnormal tissue expansion like hyperplasia, we analyzed the ratio of tissue area in relation to total lung explant area (circumference) in control and MSC-CM treated lung slices. This ratio represents a size-independent measure of tissue alterations. Notably, the ratio of the tissue to the total lung area was not changed, demonstrating a proportional growth induced by MSC-CM (Fig. 2c). We further calculated the increase of peripheral airway bud numbers from day 0 to day 2 in culture, which was significantly higher in MSC-CM-treated explants (*p* < 0.05, Fig. 2d, e). To exclude possible differences in lung explant cell composition, we conducted immunofluorescence staining of EpCAM (Fig. 2f). No obvious abnormal structures were observed in MSC-CM-treated lungs. The control lungs as well as the lungs cultured in MSC-CM showed a comparable degree of parenchymal development, which confirms the finding that MSC-CM does not cause abnormal tissue expansion. Taken together, these results support an enhanced growth and structural maturation with regard to peripheral airway buds induced by MSC-CM, in addition to the elevated functional activity of fetal alveolar epithelial cells shown above.

### Epithelial integrity and metabolic activity are not affected by MSC-CM

**Fig. 3:**
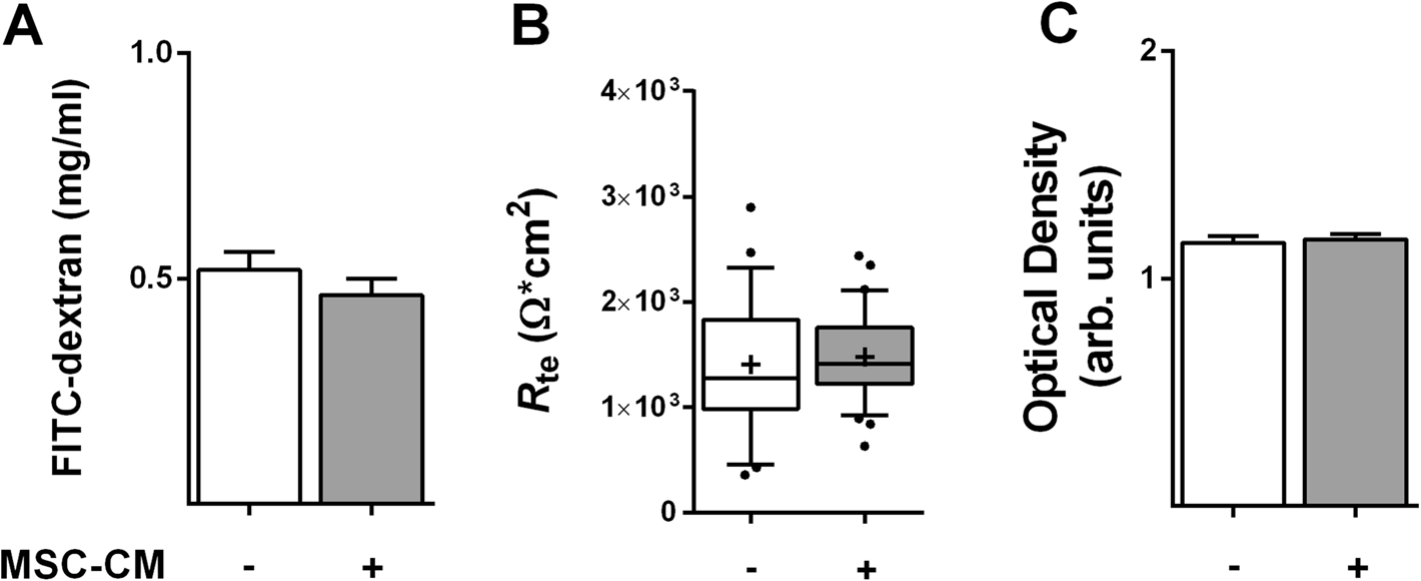
Epithelial integrity and metabolic activity of FDLE cells were not affected by MSC-CM. FDLE cells were subjected to MSC-CM or control medium for 24 h. Data are displayed as mean + SEM or box and whiskers with the 10–90 percentiles, mean (+) and median (horizontal line). Statistical differences between the two groups were analyzed with a *t* test. **a** Epithelial integrity was determined with FITC-dextran assays. MSC-CM had no effect on the permeability of FDLE monolayers (*n* = 8, 2 IE). **b** *R*_te_ was not altered by MSC-CM, determined in Ussing chambers (*n* = 28–31, 2 IE). **c** No altered metabolic activity was observed in MSC-CM-treated FDLE cells by MTT assays (*n* = 6, 2 IE). □ control, ■ MSC-CM

We analyzed whether the epithelial barrier function is altered by MSC-CM in FDLE monolayers. Epithelial permeability was determined with FITC-dextran assays, which showed that permeability of MSC-CM-treated FDLE and control epithelia were not significantly different (Fig. 3a). The *R*_te_ of monolayers was equal in the control medium and MSC-CM (Fig. 3b). Metabolic activity was assessed in MTT assays. MTT assays are generally considered to determine the proliferation rate of cells, which was not affected by MSC-CM after 24 h (Fig. 3c).

### MSC-CM-induced Na^+^ transport is not mediated by growth factors

**Fig. 4:**
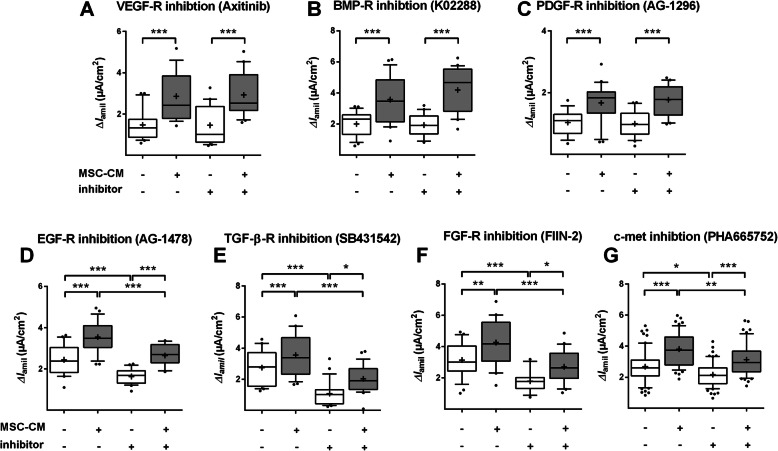
Growth factors were not responsible for the stimulating effect of MSC-CM on Na^+^ transport. FDLE cells were subjected to MSC-CM or control medium for 24 h with or without the respective growth receptor inhibitor. Data are displayed as boxes and whiskers with the 10–90 percentiles, mean (+), and median (horizontal line). Statistical differences among groups were analyzed with an ANOVA and Tukey’s post hoc test. **a** Inhibition of VEGF-R with Axitinib (*n* = 19–23, 2 IE; ***p < 0.001), **b** BMP-R with K02288 (*n* = 22–24, 2 IE; ***p < 0.001), and **c** PDGF-R with AG-1296 (*n* = 18–22, 2 IE; ***p < 0.001) did not affect Na^+^ transport in MSC-CM-treated and control cells. **d** The EGF-R inhibitor AG-1478 (*n* = 19–24, 2 IE; ***p < 0.001), **e** the TGF-β-R inhibitor SB431542 (*n* = 21–24, 2 IE; *p < 0.05; ***p < 0.001), **f** the FGF-R inhibitor FIIN-2 (*n* = 16–24, 2 IE; *p < 0.05; **p < 0.01; ***p < 0.001), and **g** the HGF-R (c-met) inhibitor PHA665752 (*n* = 64–67, 6 IE; **p < 0.01; ***p < 0.001) reduced ∆*I*_amil_ in control and MSC-CM-treated cells. Inhibition did not prevent the stimulating effect of MSC-CM. □ control, ■ MSC-CM

To determine which pathway MSC-CM increased Na^+^ transport in FDLE cells, we inhibited several growth factors by blocking their respective cellular receptor. Inhibition of the VEGF-R with Axitinib revealed that VEGF-R signaling is not involved, since MSC-CM was still able to strongly elevate ∆*I*_amil_ compared to control cells (*p* < 0.001, Fig. 4a). Similarly, inhibition of BMP-R with K02288 did not prevent the significant increase of ∆*I*_amil_ induced by MSC-CM (*p* < 0.001, Fig. 4b)*.* AG-1296, inhibiting the PDGF-R, did also not affect the stimulation of ∆*I*_amil_ induced by MSC-CM (*p* < 0.001, Fig. 4c). AG-1478, which selectively blocks the EGF-R, significantly decreased ∆*I*_amil_ in both culture conditions; however, MSC-CM was still able to significantly increase ∆*I*_amil_ compared to control cells (*p* < 0.001, Fig. 4d). This indicates that EGF-R inhibition by AG-1478 was non-specific and probably mediated by a different mechanism independent of MSC-CM components. Similar observations were obtained for the following inhibitors. The TGF-β-R inhibitor SB431542 decreased ∆*I*_amil_ in both MSC-CM-treated and control cells (*p* < 0.001, Fig. 4e). Nevertheless, SB431542 did not prevent the increased ∆*I*_amil_ induced by MSC-CM (*p* < 0.05). Furthermore, inhibition of the FGF-R by FIIN-2 decreased ∆*I*_amil_ in both, MSC-CM-treated and control cells, and MSC-CM significantly increased ∆*I*_amil_ in the presence of FIIN-2 (*p* < 0.05, *p* < 0.001, Fig. 4f). In addition, PHA665752 inhibiting the HGF receptor c-Met reduced ∆*I*_amil_ in MSC-CM-treated cells (*p* < 0.01, Fig. 4g), but MSC-CM significantly elevated ∆*I*_amil_ in the presence of PHA665752 (*p* < 0.001 Fig. 4g). Thus, none of the tested growth factor inhibitors specifically blocked the actions of MSC-CM. Therefore, the involved pathways probably did not contribute to the MSC-CM-induced stimulation of Na^+^ transport. Inhibition of the EGF-R, the TGF-β-R, the FGF-R, and c-met demonstrated a general involvement in the regulation of Na^+^ transport, while VEGF-R, BMP-R, and PDGF-R lacked any effect. Therefore, the stimulating effect of MSC-CM on Na^+^ transport was not affected by the inhibition of these growth factor pathways. As stated above, we report the equivalent *I*_SC_, calculated according to Ohm’s law. In accordance with ∆*I*_amil_, ∆*V*_amil_ confirmed the lack of growth factor contribution to the stimulated Na^+^ transport by MSC-CM (Fig. S2). *R*_te_ of the Ussing chamber measurements is also reported in the supplements (Fig. S3).

### Kinase signaling via PI3-K/AKT contributes to the MSC-mediated Na^+^ channel activity

**Fig. 5:**
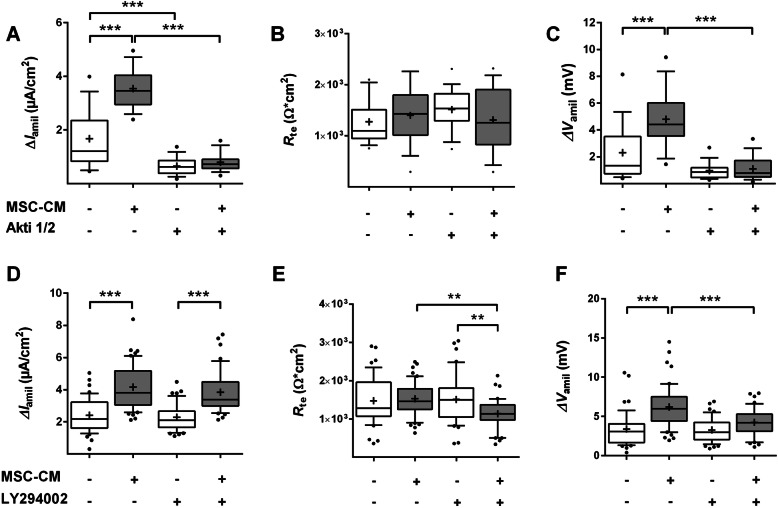
Inhibition of kinase signaling decreased MSC-CM-mediated Na^+^ channel activity. FDLE cells were subjected to MSC-CM or control medium for 24 h with or without the respective kinase inhibitor. Data are displayed as boxes and whiskers with the 10–90 percentiles, mean (+) and median (horizontal line). Statistical differences among groups were analyzed with an ANOVA and Tukey’s post hoc test. **a** ∆*I*_amil_ was significantly reduced in both, MSC-CM-treated and control FDLE cells, when Akti 1/2 was present in the culture medium. MSC-CM was not able to increase ∆*I*_amil_ anymore (*n* = 17–19, 2 IE; ***p < 0.001). **b** Akti 1/2 did not affect *R*_te_ of FDLE cells. **c** ∆*V*_amil_ of both control and MSC-CM-treated cells was decreased by Akti 1/2. AKT inhibition further prevented the increase of ∆*V*_amil_ induced by MSC-CM (***p < 0.001). **d** LY294002-mediated inhibition of PI3-K did not affect ∆*I*_amil_ in MSC-CM-treated FDLE cells (*n* = 42–50, 8 IE; ***p < 0.001). **e** LY294002 significantly decreased *R*_te_ of FDLE cells cultured in MSC-CM (**p < 0.01). **f** ∆*V*_amil_ of MSC-CM-treated cells was decreased by LY294002, which further prevented the MSC-CM-induced increase of ∆*V*_amil_ (***p < 0.001). □ control, ■ MSC-CM

Inhibition of growth factor signaling via their respective receptors did not prevent the stimulating effect of MSC-CM on Na^+^ transport. Previous studies showed that PI3-K and AKT signaling increased ENaC activity [7, 29]. We therefore determined whether any of these kinases were involved in mediating the MSC-CM effect. Incubation with MSC-CM alone strongly elevated *ΔI*_amil,_ compared to control cells (*p* < 0.001, Fig. 5a). Inhibition of AKT with Akti 1/2 significantly reduced Na^+^ transport in both, control as well as MSC-CM-treated cells compared to cells cultured without Akti 1/2 (*p* < 0.001). Notably, in the presence of Akti 1/2, MSC-CM was not able to increase Na^+^ transport anymore, suggesting a critical involvement of AKT signaling in the stimulating effect of MSC-CM. Inhibition of AKT signaling had no effect on *R*_te_ (Fig. 5b), while ∆*V*_amil_ measurements confirmed that AKT inhibition prevented the stimulating effects of MSC-CM (*p* < 0.001, Fig. 5c). AKT is activated through PI3-K signaling, which is why we also determined the contribution of the PI3-K by its inhibition with LY294002. MSC-CM incubation significantly enhanced ENaC activity in FDLE cells compared to control cells (*p* < 0.001, Fig. 5d). However, MSC-CM was also able to increase Δ*I*_amil_ in the presence of LY294002, suggesting that PI3-K signaling is not involved in the stimulating effect of MSC-CM. Indeed, there was no significant difference between MSC-CM-treated cells in the presence or absence of LY294002. However, LY294002 also strongly reduced the *R*_te_ of MSC-CM-treated FDLE cells (*p* < 0.001, Fig. 5e). As we report the calculated *I*_SC_, changes in *R*_te_ affect the resulting equivalent *I*_SC_. Indeed, *V*_te_ revealed a significant reduction of ∆*V*_amil_ in MSC-CM-stimulated FDLE cells by LY294002 (*p* < 0.001, Fig. 5f). On the other hand, ∆*V*_amil_ in control cells was not affected by LY294002 and importantly MSC-CM was not able to increase ∆*V*_amil_ in the presence of LY294002. These observations support a critical involvement of PI3-K signaling in the MSC-CM-induced Na^+^ transport. In summary, evidence suggests that both AKT and PI3-K signaling contribute to the enhanced ENaC activity induced by MSC-CM.

### Rac1 signaling contributes to the MSC-CM-mediated ENaC activity

**Fig. 6:**
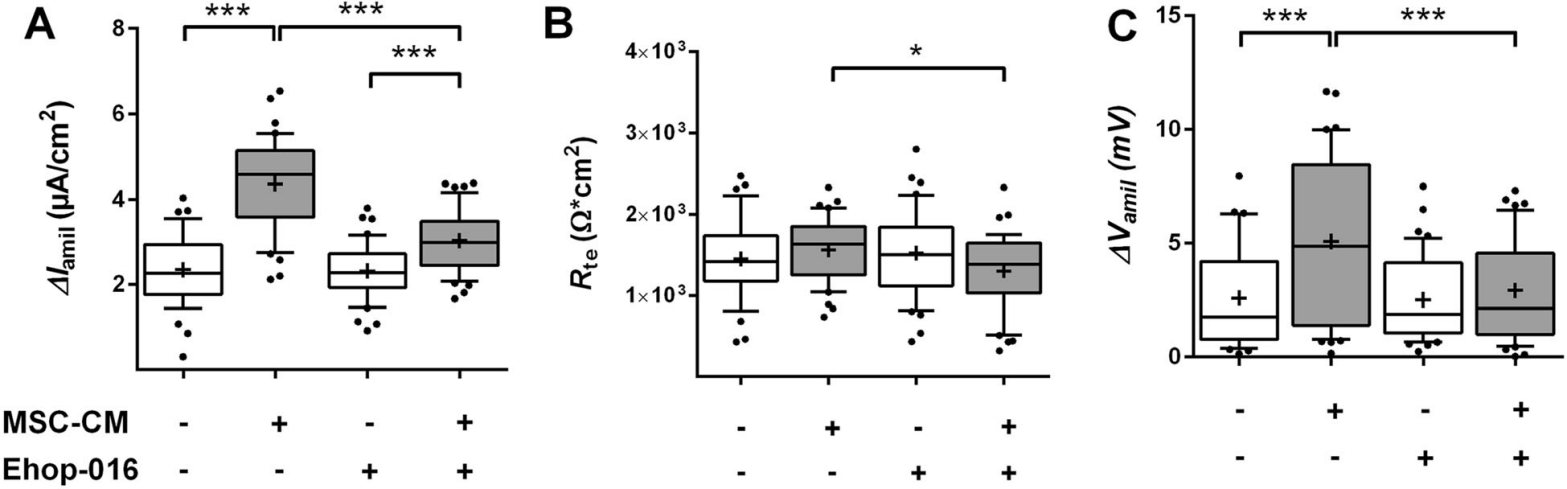
Inhibition of Rac1 impaired the effect of MSC-CM on Na^+^ transport. FDLE cells were subjected to MSC-CM or control medium for 24 h with or without the Rac1 inhibitor. Data are displayed as boxes and whiskers with the 10–90 percentiles, mean (+) and median (horizontal line). Statistical differences among groups were analyzed with an ANOVA and Tukey’s post hoc test. **a** ∆*I*_amil_ was significantly reduced by Ehop-016 in FDLE cells cultured in MSC-CM. Controls were not affected by Rac1 inhibition (*n* = 38–44, 5 IE; ***p < 0.001). **b** *R*_te_ of FDLE cells in MSC-CM was decreased by Ehop-016 (*p < 0.05). **c** ∆*V*_amil_ was decreased by Ehop-016 in MSC-CM-treated cells, which prevented the MSC-CM-induced increase of ∆*V*_amil_ (***p < 0.001). □ control, ■ MSC-CM

Another mediator activated by RTKs is Rac1, a small Rho-GTPase, that has been shown to positively regulate ENaC activity in the kidney [30]. To analyze the involvement of Rac1 signaling, we used the Rac1 inhibitor Ehop-016 and measured the MSC-CM-induced ENaC activity. MSC-CM strongly elevated ∆*I*_amil_ in the absence and presence of Ehop-016 (*p* < 0.001, Fig. 6a). Rac1 inhibition further significantly decreased ∆*I*_amil_ in MSC-CM-treated cells (*p* < 0.001). Notably, ∆*I*_amil_ in control cells was not affected by Rac1 inhibition, suggesting that Rac1 is not required for basal Na^+^ transport, but contributes to the stimulating effect of MSC-CM. However, Rac1 inhibition reduced the *R*_te_ of FDLE cells cultivated in MSC-CM (*p* < 0.05, Fig. 6b), which affects the calculated *I*_SC_. Moreover, the MSC-CM-induced increase of Na^+^ transport in the presence of Ehop-016 was not observed for ∆*V*_amil_ (Fig. 6c). ∆*V*_amil_ was strongly increased by MSC-CM and significantly reduced by the addition of Ehop-016 (*p* < 0.001). In contrast to ∆*I*_amil_, ∆*V*_amil_ was not different between control and MSC-CM-treated cells in the presence of Ehop-016. These results suggest that Rac1 signaling is involved in MSC-CM-stimulated ENaC activity, but is not solely responsible for the effect.

## Discussion

MSCs are known to secrete important growth factors, and these growth factors have been shown to play a major role in lung development and epithelial integrity (Table 1). However, little is known about the effect of MSCs and their secreted factors on functional and structural lung maturation in the context of prematurity. Our results suggest that MSC-CM improves functional and structural maturation in immature fetal lungs. MSC-CM strongly stimulated Na^+^ transport across fetal lung epithelial cells as well as Na^+^ channel mRNA expression, which has not been shown before. Furthermore, the growth and branching of fetal lung explants were accelerated by MSC-CM. Several studies have shown the beneficial effect of MSCs in rodent models of BPD, regardless of whether cells or CM were administered [56–58]. However, rodent models of BPD are treated with hyperoxia to induce oxygen toxicity which mimics only some pathological features of the disease [59, 60]. Moreover, newborn rodent pups are viable without O_2_ support and do not develop immaturity-based respiratory distress. These animal models are thus unsuitable to study the effect of MSCs on lung maturation, and it remains unknown if MSCs induce maturation in immature lung cells. To our knowledge, this is the first study demonstrating a functional improvement of fetal lung cells by MSCs. This is in contrast to previous studies in which MSCs mainly counteracted the experimentally induced damage.

**Table 1:** Effect of growth factors on lung structure and function

	Effects on the lung	Cell/tissue/organ model
**BMP-4**	↓ proliferation of the lung endoderm; inhibited outgrowth [31]	Fetal mouse lung explant
	↑ parabronchial smooth muscle cell differentiation [32]	Embryonic mouse lung explant
**EGF**	↓ ENaC activity; Na,K-ATPase activity unchanged [33]	Adult mouse collecting duct
	↑ fetal pulmonary epithelial cell growth [34]	Fetal rabbit
	↓ branching in EGF-R deficient lungs [35]	Neonatal mouse lung
	↑ lung growth in hypoplastic lungs [36]	Fetal rabbit lung
**FGF-10**	Maintained alveolar epithelial cell proliferation [37]	Neonatal mouse lung
	Directed bipotent progenitors towards ATII lineage [38]	Neonatal rat lung
	↑ (short-term) Na,K-ATPase activity [39]	A549 cells
	↑ outgrowth of the lung epithelium [31]	Fetal mouse lung explant
**HGF**	involved in septal formation [40]	Mouse embryonic lung
	↓ ENaC activity [41]	Nasal epithelial cells
	↑ growth and lumen formation [42]	Fetal mouse alveolar epithelial cells
	↑ epithelial integrity (*R*_te_) [43]	CFBE41o cells
**KGF**	↑ surfactant gene expression [44]	Fetal rat alveolar epithelial cells
	↑ epithelial cell proliferation in the airway and alveoli [45]	Adult rat alveolar epithelial cells
	↑ AFC [46]	Adult rat alveolar epithelial cells
	↑ Na,K-ATPase; ↓ ENaC mRNA expression [47]	Adult rat alveolar epithelial cells
**PDGF**	alveolar myofibroblast differentiation & alveogenesis [48]	PDGF-A mutant mice
	↑ growth of mesenchymal cells during lung development [49]	Mice overexpressing PDGF-A
	↓ lung branching [50]	Embryonic mouse lung
	↑ ENaC internalization [6]	Mouse lung epithelial & A549 cells
	↑ alveolar epithelial to mesenchymal transition [51]	A549 cells
**VEGF**	↑ surfactant protein production [52]	Adult rat alveolar epithelial cells
	↑ lung angiogenesis [53]	Neonatal rat lung
	↑ growth of the distal airway epithelial cells [54]	Human fetal lung explant
	↑ SFTP-B-positive cell number in the airway epithelium [55]	Fetal sheep lung

Na^+^ transport mediated by ENaC and Na,K-ATPase plays a central role in AFC at birth [61] and is crucial for postnatal survival, as shown by mice lacking the pore-forming α-subunit of ENaC [62]. Preterm infants with RDS had reduced airway epithelial Na^+^ transport [63] and reduced ENaC expression [64] compared with preterm infants without RDS or term infants. Furthermore, the survival of adults with acute RDS (ARDS) is related to the efficiency of their AFC [65] and ARDS, i.e., as a consequence of viral infections like the novel SARS-CoV-2, is associated with high morbidity and mortality [66]. This underlines the importance of therapies that enhance AFC and lung maturation to prevent BPD or ARDS in preterm and adult patients under intensive care. Herein, we showed that FDLE cells incubated in MSC-CM display a higher ENaC and Na,K-ATPase activity as well as mRNA expression. MSCs therefore strongly stimulated functional maturation of the fetal lungs in a paracrine manner. The improvement of AFC is of high clinical relevance, and the stimulating effect of MSC-CM on Na^+^-transport adds to the previously demonstrated beneficial effects of secreted factors raising the translational potential. Similar effects of MSCs on Na^+^ transport were seen in models of acute lung injury (ALI) and organs rejected for transplantation. In detail, MSCs enhanced mRNA expression of *Na,K-ATPase-α*_*1*_ and *-β*_*1*_ subunits in a lipopolysaccharide (LPS)-induced model of ALI [46]. Furthermore, MSC-CM accelerated Na^+^ transport and epithelial integrity in a model of ALI [67]. Restored AFC was shown by MSCs and their microvesicles in the lungs that were rejected for transplantation [68, 69]. These studies are in line with our results; however, they were conducted in challenged models or diseased transplants, whereas we show a stimulating effect of MSC-CM on Na^+^ transport in undamaged fetal alveolar cells. Therefore, we can infer that MSCs and their secreted factors stimulate functional maturation of fetal lung cells independent of a noxious cell environment. In addition to Na^+^ transport, secretion of surfactant by mature ATII cells is crucial for perinatal lung transition. FDLE cells cultured with MSC-CM demonstrated elevated mRNA levels of all three surfactant genes. This is consistent with findings that BM-MSCs enhance *Sftpb* and *Sftpc* in fetal ATII cells and that resident lung MSCs promote expression of *Sftpc* when administered in a model of ALI [70, 71].

In addition to functional maturation, we determined the structural maturation of fetal lung explants, because arrested lung development is a hallmark of BPD. We confirmed a stimulating effect of MSC-CM on the growth and branching of fetal lung explants ex vivo. Similar observations have previously been shown for placenta-derived MSCs [21]. The unchanged ratio of the tissue to the total explant area indicates that the MSC-CM-induced growth increase is not due to abnormal cell proliferation resulting in tissue hyperplasia. MSC-CM rather led to a proportional tissue growth preserving the tissue to airspace ratio. The stimulation of growth and branching in lung explants and the increase of surfactant mRNA expression confirmed an improved structural maturation of the fetal lungs. The strong effect on functional and structural maturation in the unchallenged cells and lungs shows that the factors secreted by MSCs are not only a treatment option for diseases like BPD or ALI, but also for newborns with immature lungs. To enhance the translation from bench to bedside, it is important to determine the exact factors responsible for the stimulation of functional and structural maturation in future studies. However, the maturational improvements determined in our study already show that MSCs are a powerful tool to combat respiratory diseases associated with lung immaturity or impaired AFC.

Epithelial integrity and metabolic activity were unaltered by MSC-CM. *R*_te_ of FDLE monolayers displayed tight monolayers of a high barrier function with little dextran passage even after 24 h. Under these circumstances, MSC-CM exerted no effect on barrier function or metabolic activity. In contrast, MSC-CM stimulated the proliferation and metabolic activity of A549 cells, a human lung epithelial-like cell line [72]. The differential outcome might be due to the cells itself or the used MSC-CM. A549 cells are an immortalized cancer cell line, while we used primary fetal lung epithelial cells. Moreover, the increased proliferation in A549 cells was achieved with 50-fold concentrated MSC-CM [72], in contrast to our study, where we used MSC-CM without concentration. Comparing the results obtained with lung explants to FDLE cells, 24 h MSC-CM incubation might not be long enough to induce proliferation as the growing lung explants were cultured over 4 days. The lung explants used in our study were isolated on ED15, within the pseudoglandular stage of lung development, while the FDLE cells were isolated at the late canalicular to the early saccular stage. It might be explicable that proliferation (growth) was induced by MSC-CM at earlier stages of development; while functional differentiation was induced in FDLE cells, isolated from fetal pups 24–48 h prior to birth.

A variety of studies showed that MSCs secrete important factors, e.g., growth or anti-inflammatory factors. The lung mesenchyme and its secreted paracrine factors are indispensable during lung development. After determining the stimulating effect of MSC-CM, we analyzed if specific growth factors, known to play an essential role in lung development (Table 1), are involved. Inhibition of VEGF-R, BMP-R, and PDGF-R did not affect Na^+^ transport, neither in control nor in MSC-CM-treated FDLE cells. We conclude that VEGF-R, BMP-R, and PDGF-R signaling are not contributing to the basal as well as stimulated Na^+^ transport. To our knowledge, no study has tested the influence of these growth factors on Na^+^ transport and ENaC function in fetal lung epithelial cells. EGF-R, TGF-β-R, FGF-R, and HGF-R (c-met) inhibition decreased Na^+^ channel activity in MSC-CM-treated cells and controls. We therefore assume that the reduction of Na^+^ channel activity likely represents a general effect, independent of MSC-CM. Additionally, Na^+^ channel activity was still higher in MSC-CM-treated FDLE cells compared to controls in the presence of the respective inhibitor. EGF-R, TGF-β-R, FGF-R, and HGF-R (c-met) signaling are thus not responsible for the stimulation of Na^+^ transport by MSC-CM. Previous studies reported contrary results of the EGF-mediated effect on ENaC activity. Some studies showed a stimulating effect, others demonstrated inhibition of ENaC by EGF [5, 73, 74]. We observed a decrease of Na^+^ channel activity by EGF-R inhibition that suggests EGF as a positive regulator of Na^+^ transport in FDLE cells. However, other studies of our laboratory demonstrated that EGF reduced ENaC activity (unpublished communication), suggesting that the inhibitor might affect Na^+^ transport independent of blocking EGF actions. Surprisingly, TGF-β-R inhibition lowered Na^+^ transport in FDLE cells. TGF-β has been shown to block ENaC activity in alveolar cells, and thus, receptor inhibition would be assumed to enhance Na^+^ transport [6]. On the other hand, the presence of TGF-β in MSC-CM has not been tested and the TGF-β-R inhibitor blocks not only Alk5, but also Alk-4 and Alk-7 [75], which might also affect Na^+^ transport and possibly lead to the obtained results. Key players in lung development are FGFs, e.g., FGF-10 or KGF (FGF-7) and HGF. Inhibition of FGF-R1-4 and HGF-R (c-met) lowered ENaC activity, suggesting that these growth factors have an impact on ENaC activity. However, neither FGF-R nor HGF-R inhibition prevented the stimulating effect of MSC-CM on Na^+^ channel activity. In conclusion, none of the blocked pathways was involved in the stimulating action of MSC-CM. MSCs induce Na^+^ transport either by a yet unrecognized signaling pathway or growth factors act in concert converging at later steps that are activated by more than one factor.

Since the growth factor analyses did not reveal cellular pathways leading to the enhanced Na^+^ transport, common intracellular signaling cascades were examined. These signaling cascades are activated by RTKs and stimulate Na^+^ transport, especially ENaC. PI3-K/AKT signaling pathway, e.g., activated through insulin or 17β-estradiol, was shown to stimulate ENaC activity [7, 29]. Furthermore, actin and the disruption of actin filaments influences epithelial Na^+^ channels [76]. Actin dynamics are mainly mediated by Rho-GTPases like Rac1, whose signaling pathway is also activated by RTKs and was shown to regulate ENaC in the kidney [30]. Additionally, several studies have shown convergence of these pathways, where PI3-K promoted Rac1 signaling [77, 78]. Inhibition of AKT vigorously decreased ENaC activity in control and MSC-CM-treated FDLE cells. Akti 1/2-mediated AKT inhibition completely abolished the effect of MSC-CM. Although Akti 1/2 also significantly reduced ∆*I*_amil_ in controls, this effect was not seen for ∆*V*_amil_, and ∆*V*_amil_ confirms the prevention of MSC-CM efficacy by Akti 1/2. In contrast to AKT, no effect of the PI3-K inhibitor was seen on ∆*I*_amil_ in control and MSC-CM-treated cells. On the other hand, ∆*V*_amil_ and *R*_te_ of MSC-CM-treated FDLE cells were decreased by PI3-K inhibition. The increase of Na^+^ transport induced by MSC-CM in the presence of PI3-K inhibition was not evident for ∆*V*_amil_. In this case, the reduced *R*_te_ erroneously lowered the calculated ∆*I*_amil_, making the results of ∆*V*_amil_ more reliable, which suggests that MSC-CM at least partly induced Na^+^ transport via PI3-K signaling. Notably, inhibition of Rac1 decreased Na^+^ transport in MSC-CM-treated FDLE cells that were not seen in controls. Although MSC-CM was still able to stimulate ∆*I*_amil_ in the presence of the Rac1 inhibitor, this effect was not seen for ∆*V*_amil_. Here, MSC-CM was not able to increase ∆*V*_amil_ after Rac1 inhibition, suggesting that Rac1 signaling contributes at least in part to the stimulating effect of MSC-CM on Na^+^ transport. Regarding the results obtained in our study, we propose that yet unknown growth factors secreted by MSCs activate PI3-K and/or Rac1 signaling which leads to an increased ENaC activity. Furthermore, activated PI3-K can stimulate Rac1, as shown previously [78]. However, the exact mechanisms, secreted factors, and activated pathways responsible for the stimulation of functional and structural lung maturation have to be determined in future studies prior to clinical translation. Nevertheless, it is apparent that factors secreted by MSCs harbor a therapeutic potential for stimulation of lung maturation.

## Conclusion

In conclusion, within this study, we were able to show that MSCs and their secreted factors in MSC-CM stimulate functional lung maturation by enhancing Na^+^ transport and surfactant protein mRNA expression in FDLE cells. We were also able to show that MSC-CM stimulated the structural maturation of the fetal lungs. In contrast to most studies, these results were obtained without any damaging insult like hyperoxia or LPS. Therefore, the study proposes a stimulation of maturation in the fetal lungs by MSCs instead of their repair capacity shown by others. These results thus suggest that MSCs may exert additional beneficial therapeutic effects on the immature lungs of preterm infants prior to the onset of secondary or chronic lung diseases, which may represent a novel strategy to enhance the arrested lung development.

## Supplementary Information

**Additional file 1.**

## Data Availability

All data generated or analyzed during this study are either included in this published article [and its supplementary information files] or can be obtained from the corresponding author on reasonable request.

## Abbreviations

AFC: Alveolar fluid clearance
AKT: Protein kinase B
ALI: Acute lung injury
*amil*_max_: Maximal ENaC activity
ATII: Alveolar type II
ARDS: Acute respiratory distress syndrome
BM: Bone marrow
BMP: Bone morphogenetic protein
BMP-R: Bone morphogenetic protein receptor
BPD: Bronchopulmonary dysplasia
BSA: Bovine serum albumin
CM: Conditioned medium
DAPI: 4′,6-Diamidino-2-phenylindole
DMEM: Dulbecco’s modified Eagle’s medium
ED: Embryonic day
EGF: Epidermal growth factor
EGF-R: Epidermal growth factor receptor
ENaC: Epithelial Na^+^ channel
EpCAM: Epithelial cell adhesion molecule
FDLE: Fetal distal lung epithelial
FGF: Fibroblast growth factor
FGF-R: Fibroblast growth factor receptor
FITC: Fluorescein isothiocyanate
HE: Hematoxylin and eosin
HGF: Hepatocyte growth factor
HGF-R: hepatocyte growth factor receptor (c-met)
*I*_base_: Basal current
IE: Independent experiments
*I*_SC_: Short-circuit current
LPS: Lipopolysaccharide
Mrps18a: Mitochondrial ribosomal protein S18a
MSC: Mesenchymal stem/stroma cell
MSC-CM: Mesenchymal stem cell-conditioned medium
MEM: Minimum essential media
MTT: 3-(4,5-Dimethylthiazol-2-yl)-2,5-diphenyltetrazolium bromide
NMDG: *N*-methyl-d-glucamine
*ouab*_max_: Maximal Na,K-ATPase activity
PBS: Phosphate-buffered saline
PDGF: Platelet-derived growth factor
PDGF-R: Platelet-derived growth factor receptor
PI3-K: Phosphoinositide 3-kinase
qPCR: Real-time quantitative PCR
Rac1: Ras-related C3 botulinum toxin substrate 1
RDS: Respiratory distress syndrome
*R*_te_: Transepithelial resistance
RTK: Receptor tyrosine kinase
TGF-β: Transforming growth factor beta
TGF-β-R: Transforming growth factor beta receptor
UC: Umbilical cord
VEGF: Vascular endothelial growth factor
VEGF-R: Vascular endothelial growth factor receptor
*V*_te_: Transepithelial potential
Δ*I*_amil_: Amiloride-sensitive Δ*I*_SC_
Δ*V*_amil_: Amiloride-sensitive Δ*V*_te_
