# Supplementary material for: Paracrine stimulation of perinatal lung functional and structural maturation by mesenchymal stem cells
Source: Stem Cell Res Ther. 2020 Dec 9;11:525. doi: 10.1186/s13287-020-02028-4 (PMC7724458; doi:10.1186/s13287-020-02028-4)
Supplement: Supplementary file 1 — Additional file 1. [file 13287_2020_2028_MOESM1_ESM.docx]

**Supplements**

**Isolation & characterization of human MSCs**

The study was approved by the ethical board of the medical faculty of Leipzig University. Umbilical cord (UC) tissue was collected in cold PBS (Merck, Darmstadt, Germany) with antibiotics and antimycotics after neonatal delivery or cesarean section with informed consent of mothers. After removal of cord blood, the UC tissue was washed twice in PBS and then transferred into a sterile petri dish. Afterwards the UC was cut into 3-4 cm long pieces. To remove UC vein and arteries the pieces were cut lengthways and blood vessels were pulled out using curved forceps. The remaining tissue was transferred into a tissue culture plate and chopped into pieces (<1 mm) using a scalpel. After a drying time of approximately 10 min the tissue was covered with DMEM (low glucose GlutMAX™; Life Technologies, Carlsbad, USA) supplemented with 10 % FBS (Biochrom, Berlin, Germany), penicillin (100 U/ml, Merck), streptomycin (100 µg/ml, Merck) and amphotericin B (0.25 µg/ml, Merck) and cultured at 37 °C with 5 % CO_2_. The first medium change was done after five days in culture, followed by medium exchange every three days. After approximately two weeks in culture, colonies of outgrown MSCs were detached with 0.25 % trypsin (Merck) and seeded in cell culture flask for further cultivation and passaging. Passaging of MSCs was conducted at a maximum cell confluence of 70 %. After four passages, MSC-CM was obtained from MSC cultures with a confluence of 70 % by changing the medium to DMEM (low glucose) with 2 % FBS, followed by a 72 h incubation after which MSC-CM was harvested, sterile filtrated and stored at -30 °C for further use.

MSC cell characterization was done by flow cytometry and *in vitro* multi lineage differentiation towards adipocytes, chondrocytes, and osteocytes. FITC- and phycoerythrin (PE)-labeled mouse monoclonal antibodies against CD271 (Miltenyi Biotech, Bergisch Gladbach, Germany), CD90 (Miltenyi Biotech), CD45 (BD Biosciences, Franklin Lakes, USA), CD73 (BD Biosciences), CD44 (Miltenyi Biotech) and CD11b (Miltenyi Biotech) with the appropriate isotype controls were used for analyses of cell surface markers. Cells at passage two were harvested, fixed with 2 % formaldehyde (methanol-free; ThermoFisher, Waltham, USA) and labeled with the appropriate antibodies diluted in DPBS for 1 h at 4 °C. Finally, 10,000 cells were examined with a BD Accuri™ C6 flow cytometer and analyzed using the BD Accuri™ C6 software.

All differentiation experiments were conducted in 12 well plates with cells of passage three. For chondrogenic differentiation, cells were treated with DMEM (low glucose), supplemented with 10 % FBS, antibiotics, dexamethasone (10^-8^ M, Merck), Insulin-Transferrin-Selenium (ITS; ThermoFisher), TGF-β_1_ (10 ng/ml, HEK293-derived, Peprotech, Hamburg, Germany), L-Ascorbic acid 2-phosphate sesquimagnesium salt hydrate (50 µg/ml, Merck) and linoleic acid (20 µM, Merck). Medium was changed every three days. After 14 days of differentiation, cells were fixed with 4 % formaldehyde for 15 min, washed twice with 3 % acetic acid (VWR, Radnor, USA) and stained with alcian blue 8GX (Merck). The differentiation results were determined with light microscopy.

For adipogenic differentiation cells were cultured in a 1:1 mix of “culture medium”, containing DMEM (high Glucose), 10 % FBS as well as antibiotics, and “differentiation medium”, containing DMEM (high Glucose), dexamethasone (10^-8^ M), ITS, 3-Isobutyl-1-methylxanthine (IBMX; 0.5 mM, Merck), indomethacin (0.1 mM, Merck) for the first seven days. Medium was chanced every three days. After seven days, “differentiation medium” was used exclusively. After 14 days of differentiation, cells were fixed with 4 % formaldehyde for 15 min at room temperature, washed twice with water and 60 % isopropyl alcohol prior to staining with Oil red O solution (0.45 %; Merck) for 20 min. After several washing steps with PBS, cellular red-stained lipid droplets were determined.

For osteogenic differentiation, cells were cultured in DMEM (low glucose GlutMAX™) supplemented with 10 % FBS, antibiotics, dexamethasone (10^-8^ M) and L-Ascorbic acid 2-phosphate sesquimagnesium salt hydrate (50 µg/ml) for 28 days, with a medium exchange every three days. For determination of osteoblasts, cells were fixed with 70 % ethanol and alkaline phosphatase 1 (ALP1) was stained using ALP 1 solution containing 10 ml Tris/HCl solution (1 M, pH 8.0; Merck), 1 ml N,N-dimethylformamide (VWR), 39 ml H_2_O, naphthol AS-BI phosphate (0.02 mg/ml, Merck), and Fast Red B tetrafluoroborate salt (1 mg/ml, Merck). Red-stained osteoblasts were documented microscopically.

**
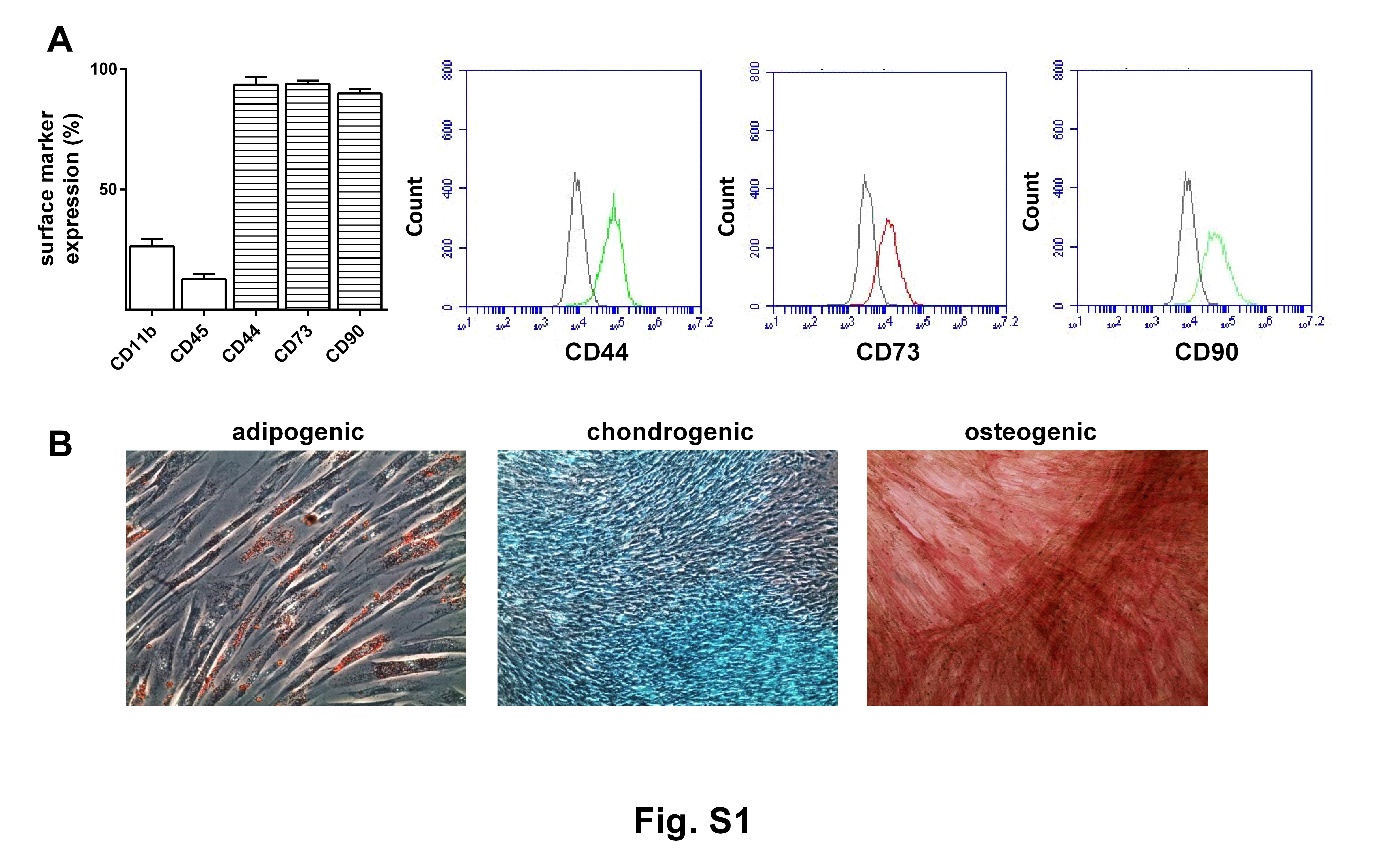
**

**Fig. S1: Characterization of UC-derived MSCs. (A)** Expression of MSC surface markers was determined by flow cytometry (n = 12; mean + SEM). **(B)** Tri-lineage differentiation into adipogenic, chondrogenic and osteogenic cells.


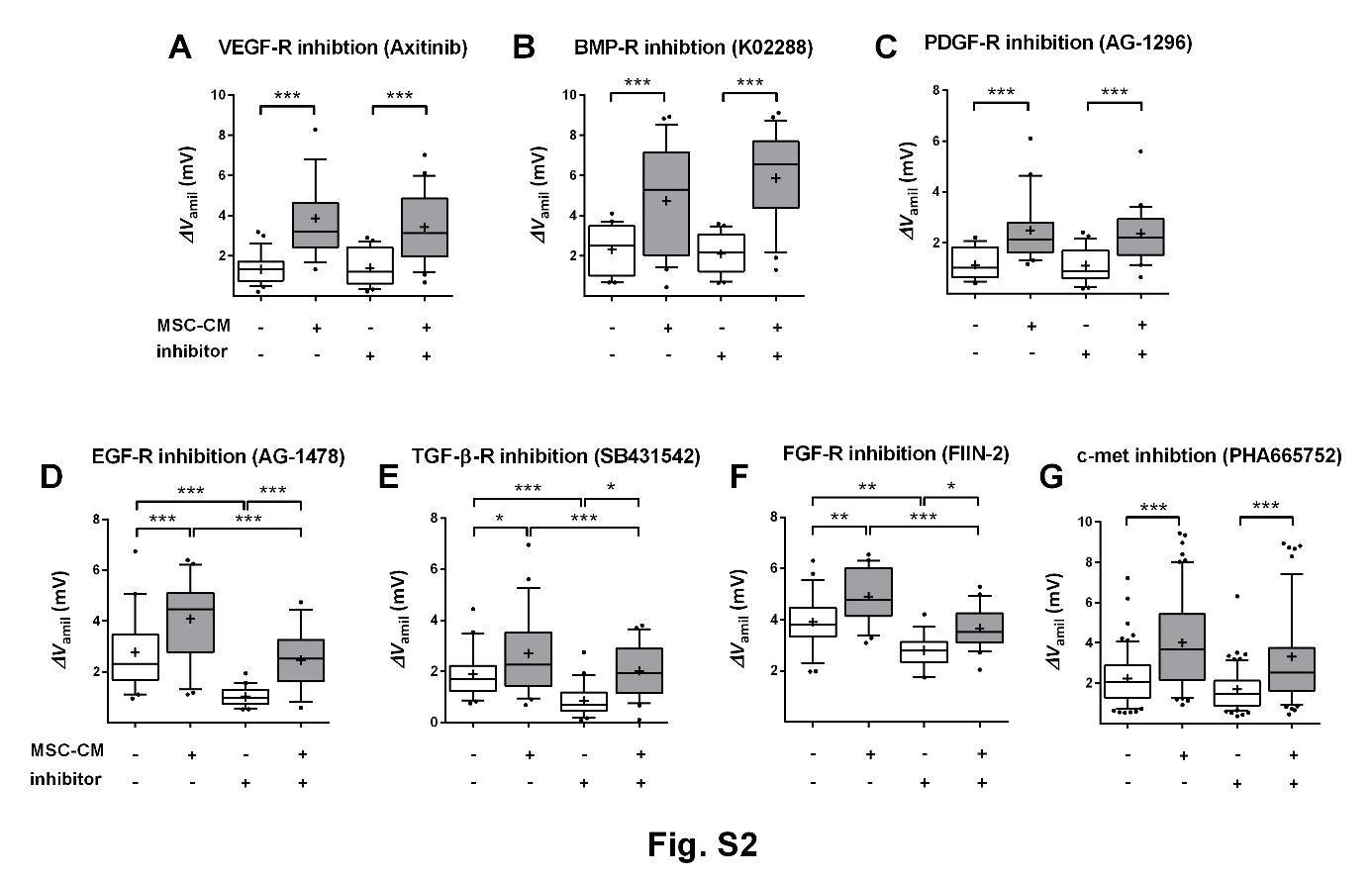


**Fig. S2: Growth factors were not responsible for the stimulating effect of MSC-CM on Na^+^ transport (∆*V*_amil_).** FDLE cells were subjected to MSC-CM or control medium for 24 h with or without the respective growth receptor inhibitor. Data are displayed as box and whiskers with the 10‑90 percentile, mean (+) and median (horizontal line). Statistical differences among groups were analyzed with an ANOVA and Tukey’s *post hoc* test. **(A)** Inhibition of VEGF-R with Axitinib (n = 19‑23, 2 IE; ***p<0.001), **(B)** BMP-R with K02288 (n = 22‑24, 2 IE; ***p<0.001) and **(C)** PDGF-R with AG-1296 (n = 18‑22, 2 IE; ***p<0.001) did not affect ∆*V*_amil_ in MSC-CM-treated and control cells. **(D)** The EGF-R inhibitor AG-1478 (n = 19‑24, 2 IE; ***p<0.001), **(E)** the TGF-β-R inhibitor SB431542 (n = 21‑24, 2 IE; ***p<0.001) and **(F)** the FGF-R inhibitor FIIN-2 (n = 16‑24, 2 IE; **p<0.01; ***p<0.001) reduced ∆*V*_amil_ in control and MSC-CM-treated cells. **(G)** The HGF-R (c-met) inhibitor PHA665752 did not affect ∆*V*_amil_ in MSC-CM-treated and control cells (n = 64‑67, 6 IE ***p<0.001). Inhibition did not prevent the stimulating effect of MSC-CM. (□) control, (■) MSC-CM.


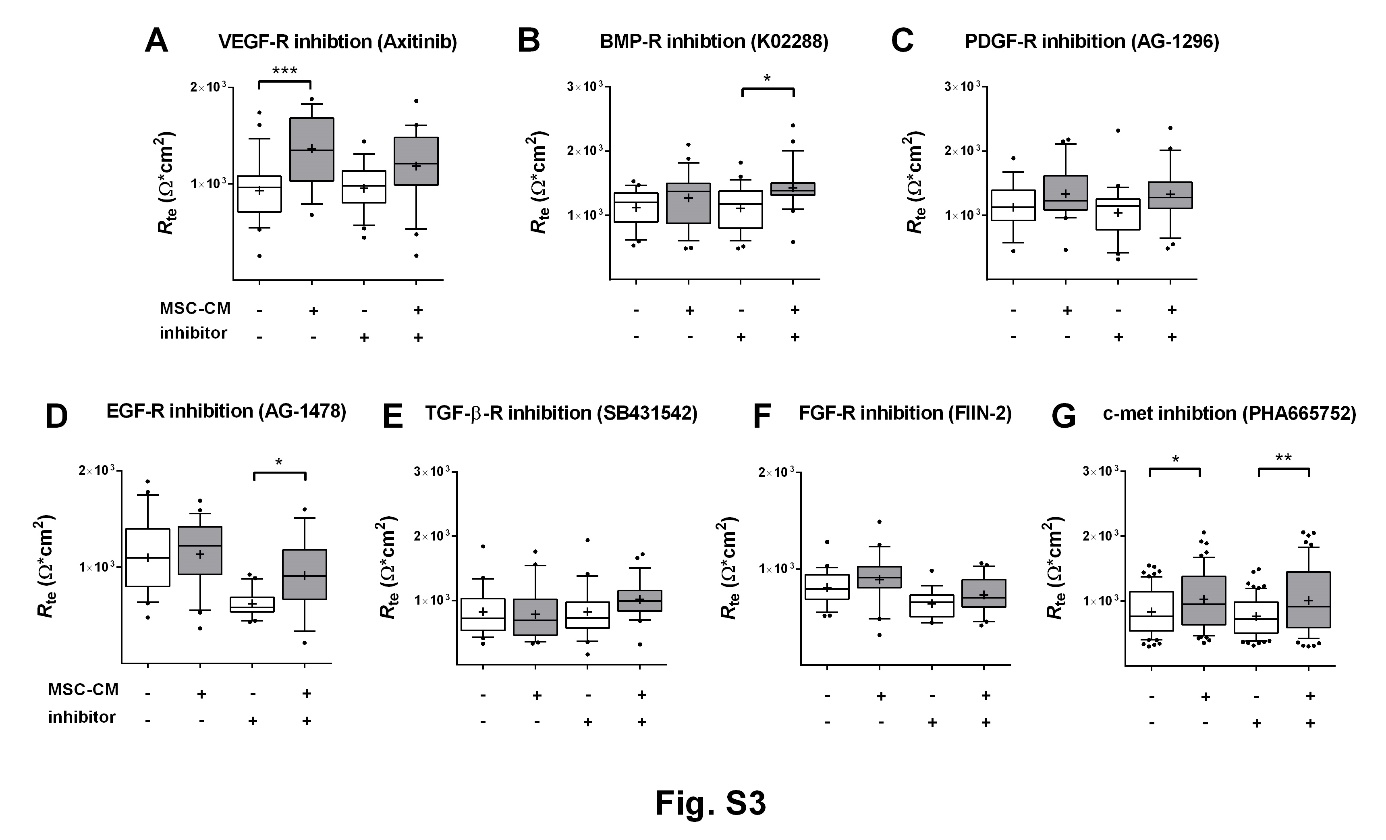


**Fig. S3: Effect of growth factor receptor inhibition on *R*_te_ of FDLE cells.** FDLE cells were subjected to MSC-CM or control medium for 24 h with or without the respective growth receptor inhibitor. Data are displayed as box and whiskers with the 10‑90 percentile, mean (+) and median (horizontal line). Statistical differences among groups were analyzed with an ANOVA and Tukey’s *post hoc* test. **(A)** The VEGF-R inhibitor Axitinib (n = 19‑23, 2 IE; ***p<0.001), **(B)** the BMP-R inhibitor K02288 (n = 22‑24, 2 IE; *p<0.05), **(C)** the PDGF-R inhibitor AG-1296 (n = 18‑22, 2 IE), **(D)** the EGF-R inhibitor AG-1478 (n = 19‑24, 2 IE; *p<0.05), **(E)** the TGF-β-R inhibitor SB431542 (n = 21‑24, 2 IE), **(F)** the FGF-R inhibitor FIIN-2 (n = 16‑24, 2 IE), and **(G)** the HGF-R (c-met) inhibitor PHA665752 (n = 64‑67, 6 IE; p<0.05; **p<0.01) did not affect *R*_te_ in MSC-CM-treated and control cells. (□) control, (■) MSC-CM.

**Analysis of total lung area**

The following macro was created with Image J (National Institutes of Health, Bethesda, USA) and saved as ijm-file:

open(); run("8-bit"); setAutoThreshold("Otsu"); setThreshold(0, 191); setOption("BlackBackground", false); run("Convert to Mask"); setTool("wand"); waitForUser("select lung outlines for measurements"); run("ROI Manager..."); roiManager("Add"); roiManager("Select", 0); roiManager("Measure"); close();

**Analysis of tissue area:**

The following macro was created with Image J (National Institutes of Health, Bethesda, USA) and saved as ijm-file:

open(); run("8-bit"); setAutoThreshold("Huang"); setThreshold(0, 159); run("Convert to Mask"); run("Measure");
